# Factors Influencing the Adoption of Online Health Consultation Services: The Role of Subjective Norm, Trust, Perceived Benefit, and Offline Habit

**DOI:** 10.3389/fpubh.2019.00286

**Published:** 2019-10-04

**Authors:** Zepeng Gong, Ziqiang Han, Xudan Li, Chao Yu, Jan D. Reinhardt

**Affiliations:** ^1^Institute for Disaster Management and Reconstruction, Sichuan University, Chengdu, China; ^2^West China School of Public Health and West China Fourth Hospital, Sichuan University, Chengdu, China; ^3^School of Political Science and Public Administration, Shandong University, Qingdao, China; ^4^School of Public Administration, Sichuan University, Chengdu, China; ^5^Swiss Paraplegic Research, Nottwil, Switzerland; ^6^Department of Health Sciences and Health Policy, University of Lucerne, Lucerne, Switzerland

**Keywords:** online health consultation service, adoption, subjective norm, habit, extended valence framework

## Abstract

The cyberspace plays an important role in improving the quality, equity, and efficiency of health services. Studying people's adoption of online health services, such as online health consultation services (OHCS) can benefit both industry and policy in the health service sector. This paper investigates influencing factors and paths of people's intention of adopting OHCS by employing the extended valence framework, with our new contribution of integrating subjective norm and offline habit into the model. Five hundred forty-three university students participated in the survey. Structural equation models and Sobel-Goodman tests were applied to test the models. The results show that subjective norm (β = 0.077, *p* = 0.041), trust in providers (β = 0.194, *p* = 0.002) and perceived benefit (β = 0.463, *p* < 0.001) positively affect the intention to adopt OHCS, while offline habit (β = −0.111, *p* = 0.026) has a negative effect. However, the association of perceived risk (β = −0.062, *p* = 0.315) and adoption is not supported. Moreover, trust in providers plays a mediating role between subjective norm and the intention of adopting, while perceived benefit mediates the relationship between trust in providers and the intention of adopting. This study highlights the importance of trust, subjective norm, perceived benefit, and persisting habits in promoting the adoption of OHCS.

## Introduction

Information and communication technologies (ICTs) have been widely used to support and to deliver health services recently (1) because ICTs' products [e.g., health information systems (HIS)] can improve the quality, efficiency, and equity of health care services delivery (2). For example, online health services (OHS), as one health information system, comprise online health consultation services (OHCS), information seeking services, and online health forums. OHS can provide health services to anyone with medical demands online at any time (3) and can overcome geographic constraints to provide services for users far away from health institutions (4). Thus, OHS has been deemed as an extension of traditional offline health services (3). With the characteristics of vast territory and inequality of socioeconomic development in China, OHS has been proposed by the Chinese government as an important option of improving the equity and efficiency of health services in China (5, 6). Meanwhile, the OHS industry is emerging and companies, such as “Hao Daifu Zaixian,” “Clove Doctor,” or “Chun Yu Doctor,” have been started, widely advertised, and being noted by the public.

Nevertheless, the public's adoption of OHS is still relatively low in China as compared with more developed countries (7). For example, the 39th Statistical Report on the Development of Chinese Internet Network showed that only 10.8% of Chinese Internet users sought health information online in 2016 (8). In comparison, 72% of U.S. adults who use the internet had searched online for health information in 2012 (9). Furthermore, a study found that less than only one percent of the visitors of the famous Chinese OHS company called “Hao Daifu Zaixian” consulted with doctors online (3). Obviously, there are still shortcomings in Chinese OHS, especially in OHCS. OHCS can provide a new channel to promote the public to obtain more diagnostic information and manage themselves and their families' health, and thereby improve the quality of health care. Meanwhile, growth in OHCS usage can further strengthen the development of the OHS industry. However, the low usage rates of OHCS may make it difficult to exert a significant effect on health services. Therefore, understanding the reasons for such low usage rate will be useful for service providers in formulating strategies aimed at increasing OHCS adoption.

OHS research papers account only for a small proportion of HIS research, based on our literature research in PubMed and Web of Science. Most of the existing literature on HIS focuses on the implementation of HIS, such as Clinical Information System, Computerized Physician Order Entry System, Electronic Health Records or Electronic Medical Record, in health institutions. For example, the determinants of adoption of these HIS haven been examined from the perspective of physicians (10, 11), nurses (2, 12), and medical institutions (13, 14). Some other studies explored how to design a HIS (15, 16) or evaluated the implementation of a HIS (17, 18).

Furthermore, among the OHS literature, research on individuals' adoption of OHCS is very limited. Although some studies have examined the factors that influence consumers to adopt OHS, most of them explored the issue of health information seeking. Trust, perceived risk, perceived usefulness, and perceived health condition are common factors that were found to influence consumers' information seeking behaviors in these studies (4, 19, 20). However, information seeking and online consultations are two related but different behaviors because information seeking is usually unilateral, but online consultation involves interaction between consumers and doctors. Indeed, several articles explored behaviors toward a non-specific comprehensive OHS, which included information seeking, online registration, online consultation, and patient education rather than the information seeking alone (3, 7, 21). No studies that solely concentrated on OHCS were identified in our literature reviews, and thus we developed the present study to contribute to closing this gap.

This study focuses on the adoption of OHCS from the perspective of the decision-making process. OHS is the application of e-commerce in the healthcare context (7), so the adoption of OHCS, as a part of OHS usage, can be considered as an e-commerce behavior. Previous studies have found that perceived benefit and risk, as well as trust are important determinants in consumers' decision-making process of purchase and adoption of online services (19, 22–25). Hence, the extended valence framework (26) including benefit perception, risk perception, and trust was adopted to guide this research. Moreover, using OHCS involves changes in habits of receiving offline medical services (3), and subjective norms are also essential for behavioral change of consumers (27). We this integrated subjective norm and offline habit in the extended valence framework. In short, this paper explores motivational and inhibiting factors of OHCS adoption to improve our current understanding of consumers' decision-making processes regarding OHCS.

## Theoretical Foundation and Hypotheses

### The Extended Valence Framework

The theoretical foundation of this study is the extended valence framework. The valence framework is a well-established theory deriving from economic and psychological theories in behavioral research (28, 29). Perceived risk and benefit are the two foundational aspects regarding consumers' decision-making and consumers tend to maximize the positive value (perceived benefit) and minimize the negative impacts (perceived risk) of a product or service when they make decision of purchasing it according to the valence framework (30, 31). The valence framework performs well in understanding individual's behavior because by considering both negative and positive attributes in decision-making simultaneously (32).

**Figure 1:**
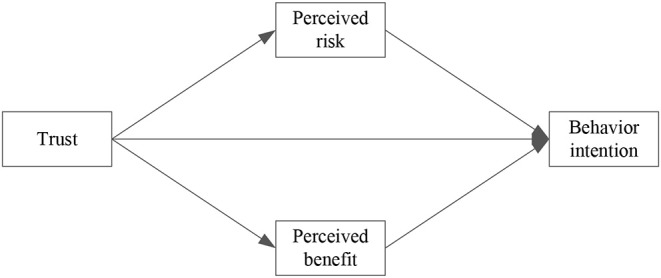
Extended valence framework.

Kim developed the extended valence framework by integrating trust into the valence framework (26). The extended valence framework states that trust, perceived risk and perceived benefit directly affect consumers' purchase intention in one way, and in an indirect way, trust influences the purchase intention through perceived risk and perceived benefit (Figure 1). The extended valence framework has been applied in studies regarding e-commerce, and has been considered a useful and valid theoretical framework to guide our understanding of people's behaviors in the e-commerce context (33), including online health information seeking behavior (4). Moreover, a benefit-risk evaluation may help strategy-planners to make more updated, informed, and effective management decisions (34). Therefore, the extended valence framework is adopted in this paper to promote the understanding of the individual's adoption of OHCS.

### Hypotheses

#### Perceived Risk

Perceived risk is considered as a salient inhibitor of the adoption of online services, because individuals may experience certain levels of risk due to the uncertainty and uncontrollability of the cyberspace (26). Perceived risk is a multifaceted concept (24) and varies according to types of products or services (35). In the context of this study, perceived risk refers to privacy and security risk concerns. Privacy and security risk involves the collection, use, and disclosure of personal information (36), as well as uncertain consequences of actions based on information and guidance provided by an online doctor (37). Health information technology may aggravate individuals' privacy concerns over the potential misuse of personal health information (38). Empirical findings have shown that there is a negative association between online privacy and security concerns and the use of online health services (21, 39). Thus, we hypothesize:

**H1**. Perceived risk has a negative effect on the adoption of OHCS.

#### Perceived Benefit

Perceived benefit is defined as a consumer's belief about the extent to which he/she will become better off through the use of certain online service (31). Evidence from prior studies has shown that perceived benefit exerted a positive and significant effect on customers' behavioral intention (23, 38, 40). OHCS as one of many internet-based services which may bring about potential benefits for consumers, such as cost and time saving (41), which have been identified as relative benefits as compared with traditional offline services (28). Accordingly, if an individual perceived a higher degree of benefits, he/she would be more likely to adopt OHCS. Thus, we hypothesize:

**H2**. Perceived benefit has a positive effect on the adoption of OHCS.

#### Trust

Trust either can be defined as one's willingness to rely upon another (42), or as the belief in dependability and honesty (43). In this study, trust refers to the trust in OHCS providers which include both online platform and online doctors. Furthermore, trust in OHCS providers is defined as the individual's confidence in the provider's integrity and dependability (44), and the belief that OHCS providers have attributes that are beneficial to consumers (37). Individuals' trust is considered as one of the most important psychological factors influencing online behaviors (31). Prior studies found that there is a positive association between trust and behavioral intention (22, 26). It's likely that people with a higher degree of trust feel less uncertainty and believe that the service will improve their effectiveness in managing their health (4). Thus, trust can influence consumers' purchase decision making both directly and indirectly through perceived benefits and risks, i.e., trust can have a positive impact on perceived benefits and a negative influence on perceived risks. In sum, trust influences consumers' intention of purchasing in e-commerce both directly and indirectly through perceived risks and benefits (26). Thus, we hypothesize:

**H3**. Trust in providers has a positive effect on the adoption of OHCS.

**H4**. Trust in providers has a negative effect on the risk perception of OHCS.

**H5**. Trust in providers has a positive effect on the benefit perception of OHCS.

#### Subjective Norm

Subjective norm refers to how an individual thinks he/she should behave and how their behavior would be judged by others in a specific cultural and social environment (45). According to the Theory of Reasoned Action, individual's intention of performing a given behavior is influenced by normative beliefs—the views (whether the individual agrees or not) regarding one behavior developed by important others, such as peers, friends or relatives (46, 47). Thus, people may shape their behavioral intentions based on how they believe significant others will view their behaviors. Previous studies have found that subjective norm affects online health-related behavioral intention positively (19, 48). Therefore, we hypothesize:

**H6**. Subjective norm has a positive effect on the adoption of OHCS.

In the current study, the subjective norm is also considered to be a determinant of trust, perceived benefit, and perceived risk. Li et al. (49) found that when people do not know a system well, they may rely on the opinions of others significant to them, and then develop trust belief toward the system accordingly (49). Moreover, prior research found that subjective norm directly affects trust (50, 51). Regarding the perceived benefit and perceived risk, few studies in the online health context have simultaneously incorporated the association between subjective norm and perceived benefit, and the association between subjective norm and perceived risk. However, it has been found in other information systems related research that subjective norm influences perceived usefulness positively (52, 53) and motivates users to reveal personal information (54). In other words, subjective norm, to a certain extent, can enhance individual perception of benefits and reduce the risk perception of private information. Therefore, we hypothesize:

**H7**. Subjective norm has a positive effect on trust in providers.

**H8**. Subjective norm has a positive effect on perceived benefit.

**H9**. Subjective norm has a negative effect on perceived risk.

#### Offline Habit

Habits can shape human's decision process, and are considered as a critical factor influencing people's behavior in the e-commerce context (55). A habit is an individual's repeated, unconscious and automatic behavior (56). Studies have shown that habits may keep individual from switching to new technologies (57). Thus, people's habits of using offline service channels may have a negative effect on their intention to switch to the online service channel (28). Regarding health services, if an individual is used to visiting doctors in hospitals (offline channel), she/he would be more willing to continue to use the offline channel rather than switching to online services. Therefore, we hypothesize:

**H10**. Offline habit has a negative effect on the adoption of OHCS.

**Figure 2:**
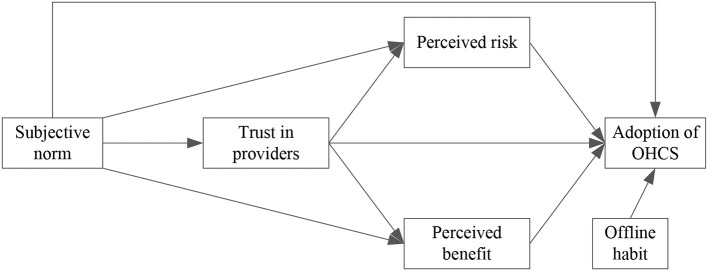
Research model.

Based on the hypotheses mentioned above, we propose our research model as depicted in Figure 2.

## Methods

### Participants and Procedures

The participants were university students, which were sampled by convenience. University students represent a large proportion of active online consumers (31), and the younger generation is the most active population participating in online transactions (58). Moreover, students younger than 30 years are the largest group of internet users, representing the majority of Internet users in China (28). Moreover, prior e-service documents including research on OHS also sampled from student populations (19, 28). Here, students from two universities in China were surveyed. We designed a web-based questionnaire, and linkage of the questionnaire was delivered to both undergraduate and graduate students via social network applications (WeChat and QQ). The questionnaire contained instructions and an explanation of the study but did not collect any information that would have made the person identifiable. The participation of each student was completely voluntary, and they could withdraw from the study at any stage. Eventually, 543 valid questionnaires were collected.

### Measurements

The adoption of OHCS (AO) was assessed by three items adapted from an instrument created by Venkatesh et al. (59). Similarly, subjective norm (SN) also included three items adapted from Venkatesh et al. (60). Trust in providers (TP) was measured by seven items (four items adapted from Cater and Bélanger (61) and Mou et al. (4) and three self-constructed items). Perceived risk (PR) was assessed by five items adapted from McKnight et al. (62) and Yi et al. (37). Perceived benefit (PB) was measured by six items including four items adapted from Mou et al. (4) and Zhang et al. (7) and two self-developed items. Offline habit (OH) was measured with four items which were adapted from Zhang et al. (7). The items are listed in Table 1. All items were assessed using five-point Likert scales ranging from strongly disagree (1) to strongly agree (5).

**Table 1:** Measurement items, factor loading, and characteristics of variables.

**Variables**	**Items**	**FL**	**Mean (SD)**	**Range**
Subjective norm	SN1: People who influence my behavior (would think/think) that I should use the OHCS.	0.875	8.39 (2.42)	3–15
	SN2: People who are important to me (would think/think) that I should use the OHCS.	0.896		
	SN3: People who are in my social circle (would think/think) that I should use the OHCS.	0.880		
Trust in providers	TP1: I would characterize OHCS providers as honest.	0.670	22.24 (3.98)	7–35
	TP2: I believe that the health service provided by OHCS platform is useful.	0.600		
	TP3: The OHCS platform performs its role as health service providers very well.	0.597		
	TP4: I have confidence in relying on OHCS platforms to complete a health consultation or diagnosis.	0.681		
	TP5: Doctors on the OHCS platform have medical qualifications.	0.794		
	TP6: The consultation or diagnosis provided by doctors on OHCS platforms is reliable	0.769		
	TP7: In my opinion, doctors on the OHCS platform are trustworthy.	0.806		
Perceived risk	PR1: Providing personal health information online is unsafe.	0.576	18.36 (3.14)	5–25
	PR2: I think it is risky to provide personal information to OHCS providers.	0.808		
	PR3: I think it is risky to provide personal health information to doctors on OHCS platforms.	0.797		
	PR4: I would hesitate to provide my personal information (such as name, address, health condition, bank information, and phone number, etc.) to OHCS platforms.	0.719		
	PR5: I think it is risky to make a decision (such as taking medicine, controlling diet, etc.) based on the diagnosis provided by the doctors on the OHCS platform.	0.631		
Perceived benefit	PB1: Using OHCS can be of benefit to me in managing my health.	0.793	21.35 3.76)	6–30
	PB2: Using OHCS can increase my knowledge of my personal health conditions.	0.804		
	PB3: Using OHCS can help to relieve stresses about my symptoms or my worries about symptoms.	0.691		
	PB4: Using OHCS will be useful for my health.	0.766		
	PB5: Compared with going to the hospital, using OHCS can save time.	0.706		
	PB6: Compared with going to the hospital, using OHCS can save medical expenses.	0.666		
Offline habit	OH1: Whenever I need to see a doctor or have a health consultation, I will choose to go to hospitals or clinics without even being aware of making another choice.	0.737	15.77 (2.50)	4–20
	OH2: Whenever I need to see a doctor or have a health consultation, I unconsciously start going to hospitals or clinics.	0.879		
	OH3: Choosing to go to hospitals or clinics when I need to see a doctor or have a health consultation is something I do unconsciously.	0.844		
	OH4: In general, I am accustomed to taking the offline channel (going to hospitals or clinics) for medical treatment or health consultation.	0.828		
Adoption of OHCS	AO1: I intend to use OHCS to consult health issues when needed in the future.	0.811	10.27 (2.01)	3–15
	AO2: I predict that I will use OHCS to consult health issues when needed in the future.	0.862		
	AO3: I plan to use OHCS to consult health issues when needed in the future.	0.845		

*FL, factor loading; SD, standard deviation*.

### Statistical Analysis

Before hypotheses testing, we first evaluated the measurement model including composite reliability, convergent validity, and discriminant validity. Then, structural equation modeling (SEM) was conducted to test the hypotheses. Goodness-of-fit indices and the path coefficients with *p*-values were reported. Based on recommendations of Wang and Lai (63), the ratio of Chi-square values (χ2) to the degrees of freedom (χ2/df) should be <3. The values of the Tucker-Lewis fit index (TLI), comparative fit index (CFI), goodness-of-fit index (GFI), and normalized fit index (NFI) should be higher than 0.9, while root-mean-square residual (RMR) and root-mean-square-error of approximation (RMSEA) should be <0.8. Besides, we conducted a mediation analysis to further examine mediating effects. Sobel-Goodman tests were applied to test the presence of mediation. Amos 21.0 was used for the SEM, and Stata/MP 14.0 was used for all other analyzes.

## Result

### Descriptive Results

Among the 543 qualified respondents, 64.27% were female; 45.12% were graduate students, and 30.94% were specialized in Medicine. Detailed results are shown in Table 2. Mean values (standard deviations) of AO (adoption of online health consultation service), SN (subjective norm), TP (trust in providers), PR (perceived risk), PB (perceived benefit), and OH (offline habit) were 10.27 (2.01), 8.39 (2.42), 22.24 (3.98), 18.36 (3.14), 21.35 (3.76), and 15.77 (2.50), respectively (Table 1).

**Table 2:** Characteristic of respondents.

**Items**		**Frequency**	**Percent (%)**
Gender	Female	349	64.27
	Male	194	35.73
Being a graduate student	No	298	54.88
	Yes	245	45.12
Major	Medicine	168	30.94
	Else	375	69.06
Experience of obtaining health information online	No	189	34.81
	Yes	354	65.19
Expense	<1,000	100	18.42
	1,000–2,000	361	66.48
	2,001–3,000	55	10.13
	More than 3,000	27	4.97
Health condition (Frequency of seeing a doctor)	Never	18	3.31
	Rarely	205	37.75
	Sometimes	224	41.25
	Usually	96	17.68

### Measurement Model Evaluation

The measurement model aims to assess reliability and validity. We used Cronbach's alpha (CA) and composite reliability (CR) to evaluate the construct reliability. The results of all CA test ranged from 0.788 to 0.914, and CR values ranged from 0.835 to 0.915 (Table 3). Since both CA and CR for each construct were higher than the minimum cutoff value of 0.7 (64), construct reliability was supported.

**Table 3:** Validity and reliability of variables.

	**CA**	**CR**	**AVE**	**SN**	**TP**	**PR**	**PB**	**OH**	**AO**
SN	0.914	0.915	0.781	**0.884**					
TP	0.907	0.874	0.500	0.467	**0.707**				
PR	0.788	0.835	0.507	−0.190	−0.318	**0.712**			
PB	0.887	0.878	0.547	0.326	0.620	−0.170	**0.740**		
OH	0.847	0.894	0.678	−0.149	−0.073	0.314	0.024	**0.823**	
AO	0.886	0.878	0.705	0.331	0.500	−0.207	0.501	−0.131	**0.840**

*CA, Cronbach's alpha; CR, composite reliability; AVE, average variance extracted. The bold diagonally are the square root of AVE*.

Concerning validity, we assessed both convergent validity and discriminant validity. Convergent validity was assessed by factor loadings and average variance extracted (AVE). The factor loadings and AVE of all constructs were higher than the suggested value of 0.5 (65), indicating a good convergent validity. Additionally, the square root of AVE for each construct was higher than its correlation coefficient with any other construct. Therefore, discriminant validity was acceptable.

### Structural Model Evaluation

**Figure 3:**
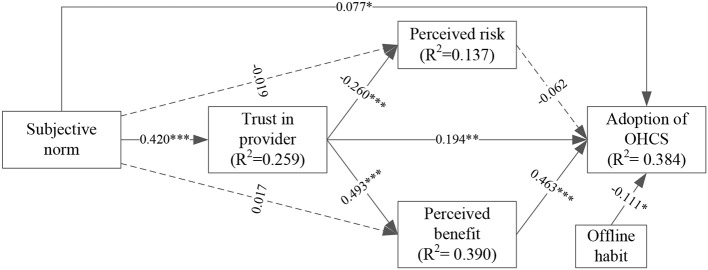
The structural model and R^2^ values. dashed lines represent unsupported paths. *p < 0.05, **p < 0.01, ***p < 0.001.

We first tested the fitness index of the structural model. The result showed that the structural model had a good fit (χ2 = 802.096, χ2/df = 2.416, RMR = 0.040, RMSEA = 0.051, GFI = 0.901, NFI = 0.916, TLI = 0.942, CFI = 0.945). Results of hypotheses testing and the SEM are shown in Table 4 and Figure 3, respectively. Perceived benefit (β = 0.463, *p* < 0.001) significantly and positively influenced the adoption of OHCS, while the effect of perceived risk (*p* = 0.315) on the adoption of OHCS was not statistically significant. Trust in providers significantly and positively affected the adoption of OHCS (β = 0.194, *p* = 0.002) and perceived benefit (β = 0.493, *p* < 0.001), but negatively affected perceived risk (β = −0.260, *p* < 0.001). Subjective norm was positively correlated with the adoption of OHCS (β = 0.077, *p* = 0.041) and trust in providers (β = 0.420, *p* < 0.001). Nevertheless, none of its effects on perceived benefit (*p* = 0.593) or on the perceived risk (*p* = 0.570) was statistically significant. Moreover, offline habit (β = −0.111, *p* = 0.026) affected the adoption of OHCS negatively. In sum, perceived benefit, trust in providers, subjective norm and offline habit had a significant direct effect on the adoption of OHCS, and the structural model could explain 38.4% of the variation in OHCS adoption (R^2^ = 0.384).

**Table 4:** Path coefficients and the result of hypotheses test.

**Hypotheses**	**Path coefficients**	**Sig**.	**Findings**
H1: PR→AO	−0.062	*P* = 0.315	Not supported
H2: PB→AO	0.463	*P* < 0.001	Supported
H3: TP→AO	0.194	*P* = 0.002	Supported
H4: TP→PR	−0.260	*P* < 0.001	Supported
H5: TP→PB	0.493	*P* < 0.001	Supported
H6: SN→AO	0.077	*P* = 0.041	Supported
H7: SN→TP	0.420	*P* < 0.001	Supported
H8: SN→PB	0.017	*P* = 0.593	Not supported
H9: SN→PR	−0.019	*P* = 0.570	Not supported
H10: OH→AO	−0.111	*P* = 0.026	Supported

### *Post-hoc* Analysis

Based on the result of the structural model, we further conducted a *post-hoc* analysis to test the mediating effect of trust in providers in the relation between subjective norm and the adoption of OHCS, and to test the mediating effect of perceived benefit in the relation between trust in providers and the adoption of OHCS. We confirmed a significant indirect effect of the subjective norm (β = 0.172, *p* < 0.001) on adoption through trust in providers, which accounted for 61.6% of the total effect that subjective norm had on adoption (Table 5). Moreover, a significant indirect effect of trust in providers (β = 0.093, *p* < 0.001) on adoption through perceived benefit was also confirmed, accounting for 36.9% of the total effect.

**Table 5:** Mediation result.

		**B**	**S.E**.	**Sobel z (% of total effect)**
Mediation of TP	Direct effect			
	SN	0.107^**^	0.035	
	TP	0.221^***^	0.021	
	Indirect effect			7.961 (61.6%)
	SN on AO through TP	0.172^***^	0.022	
Mediation of PB	Direct effect			
	TP	0.159^***^	0.023	
	PB	0.159^***^	0.025	
	Indirect effect			6.078 (36.9%)
	TP on AO through PB	0.093^***^	0.015	

*Gender, major, being a graduate student, experience of obtaining health information online, monthly expense and health condition were included in the analysis but not reported in this table*.

** *p < 0.01*,

*** *p < 0.001*.

## Discussion

This study enriches the extended valence framework by integrating subjective norm and offline habit into the extended valence framework in order to investigate the facilitators and barriers of OHCS adoption. To the best of our knowledge, this is the first study about people's decision-making regarding the adoption of OHCS in China. Our results show that trust in providers, perceived benefit, subjective norm, and offline habit significantly affect the adoption of OHCS, while the impact of perceived risk on adoption is not supported. 38.4% variation of the adoption of OHCS is explained by our proposed model, and there are four important findings.

First, the extended valence framework can partly interpret the adoption of OHCS. Perceived benefit, as expected, influences the adoption of OHCS positively—students with a higher degree of perceived benefit are more likely to adopt the OHCS. Also, consistent with existing literature (4, 19), trust in providers has a positive effect on the adoption of OHCS. Moreover, such positive effect is partly mediated by perceived benefit. Therefore, both perceived benefit and trust in providers are critical determinants of OHCS usage.

However, the association between perceived risk and the adoption of OHCS is not significant in this study. Prior studies on OHS have found that perceived risk had a negative effect on behavioral intention toward using health service (7, 39). One possible reason is that online health consultation behavior is different from other OHS behaviors (e.g., health information seeking). Investigations in South Africa and China found that perceived risk plays a role in dampening people's online health information acceptance behavior (4) or intention to use comprehensive OHS (7). The inconsistent results indicate that there is a need to conduct more research specifically focused on OHCS. Another possible explanation could be that the respondents are university students, who have a strong perception of control in online behavior. This makes them believe that they can identify potential risks and avoid risky behavior when they adopt OHCS.

This study confirms a positive associations between subjective norm and the intention of using OHCS, which is consistent with previous findings related to e-services (e.g., e-banking) (27, 66). Compared with other studies that only analyzed the direct relationship between subjective norm and intention of adoption (48, 67), we further found that trust in providers mediated the association of subjective norm and OHCS adoption. In other words, subjective norm directly influences OHCS adoption, but also influences trust in providers first, and then influences OHCS adoption indirectly through trust in providers. Obviously, both direct and indirect effect of subjective norm on the adoption of OHCS show that subjective norm must be taken into account when promoting and implementing OHCS. Unfortunately, associations of subjective norm with perceived benefit and perceived risk are not supported, and such associations deserve to be further explored.

Offline habit is the only negative factor that influences the adoption of OHCS. Habit is considered as an automatic behavioral process, which makes individuals do not evaluate the benefit and cost of their ongoing behavior (68). Hence, habitual behavior causes a lack of motivation to change ongoing behavior (7). Individuals who are used to offline health services were reluctant to transfer to OHS (28). This indicates that the low usage rate of OHCS in China may be due to people's habit of accessing medical services offline. Despite the rapid development of information and communication technology, traditional offline channels (going to hospitals or clinics for medical services) are still the first choice for people to see a doctor. Therefore, OHCS providers should take positive strategies to encourage potential consumers to adopt online services and eliminate the adverse effects of offline habits gradually (28), e.g., by enhancing individual eHealth literacy through educational programs (1).

This study has some limitations. First, the measurement of perceived risk only consisted of privacy and security concerns, while online risk should contain more facets. This implies that H1 can only reflect the non-significant association of privacy and security risk with the adoption of OHCS. Second, the respondents of the present study were only university students, and generalizations to other potential OHCS consumer groups cannot be made. Thus, studies with more representative samples should be conducted in the future. Third, this study studied behavioral intention rather than actual behavior regarding OHCS adoption. It may thus suffer from information bias, since intention and behavior are closely related but not necessarily equivalent.

## Conclusion

This study explored factors influencing of the adoption of OHCS by integrating offline habit and subjective norm into the extended valence framework. Subjective norm, trust in providers and perceived benefit were found to play promoting roles regarding individuals' OHCS adoption behavior, while habit had an inhibitory effect. The habit of using offline channels to obtain health services can play an important role in explaining low usage rate of OHCS in China. To promote the adoption of OHCS, the negative effect of offline habit should be considered besides the strengthening of trust, subjective norms and perceived benefits.

## Data Availability Statement

The datasets generated for this study are available on request to the corresponding author.

## Author Contributions

ZG, ZH, XL, CY, and JR have made a direct, intellectual contribution to this study. ZG and ZH designed and wrote most part of the paper and conducted the data analysis. XL, CY, and JR were major contributors of data collection and paper revision. All authors have read and approved the final version.

### Conflict of Interest

The authors declare that the research was conducted in the absence of any commercial or financial relationships that could be construed as a potential conflict of interest.
